# Improvement of Oral Intake after Treatment Using Enteral Feeding Tube for Large Advanced Gastric Cancer Invading Proximal Stomach: A Case Series of 20 Patients

**DOI:** 10.70352/scrj.cr.24-0143

**Published:** 2025-02-07

**Authors:** Koichi Hayano, Yoshihiro Kurata, Yasunori Matsumoto, Ryota Otsuka, Nobufumi Sekino, Takeshi Toyozumi, Akira Nakano, Tadashi Shiraishi, Masaya Uesato, Gaku Ohira, Hisahiro Matsubara

**Affiliations:** Department of Frontier Surgery, Chiba University Graduate School of Medicine, Chiba, Chiba, Japan

**Keywords:** gastric cancer, enteral feeding tube, S-1, enteral nutrition

## Abstract

**INTRODUCTION:**

Patients with large Stage IV gastric cancer (GC) invading the proximal stomach find it difficult to receive not only bypass surgery but also S-1-based chemotherapy. This study aimed to show our treatment results for those GC patients using elementary diet (ED) tubes, which enabled S-1-based chemotherapy and nutrition support.

**CASE PRESENTATION:**

We evaluated 20 patients (13 men and 7 women; median age 70 years) with large Stage IV GCs (8.7–21.9 cm) invading the proximal stomach, who were admitted due to inability to eat, treated with S-1-based chemotherapy using an ED tube. The duration from the initiation of the chemotherapy to the improvement of oral intake, changes in nutritional status, and disease-specific survival (DSS) were retrospectively investigated. Two of the 20 patients failed to complete even one cycle of chemotherapy due to severe nausea or diarrhea. The other 18 patients improved oral liquid intake after 47.5 ± 18.8 days, and 17 patients improved oral solid food intake after 54.5 ± 19.6 days from the start of chemotherapy. In addition, three patients (16.7%) could receive conversion surgery after improvement of oral intake. The median DSS of those 18 patients was 13.1 months. Serum albumin level and prognostic nutritional index (PNI) were significantly improved after about 1 month of the treatment (both *P* <0.0001). Improvement of serum albumin level and PNI during the first 1 month of the treatment significantly correlated with better DSS (*P* = 0.006, 0.01, respectively).

**CONCLUSIONS:**

Given a high oral intake success rate, S-1-based chemotherapy using an ED tube can be a promising treatment option for large Stage IV GC with poor oral intake.

## Abbreviations

DSS
: disease-specific survival
ED
: elementary diet
GC
: gastric cancer
PNI
: prognostic nutritional index
SOX
: S-1 plus oxaliplatin

## INTRODUCTION

Gastric cancer (GC) is the third leading cause of cancer-related death and is the fifth most common malignancy.^1)^ The current standard treatment strategy for stage IV GC is systemic chemotherapy. A phase II trial of first-line chemotherapy with S-1 plus oxaliplatin (SOX) yielded promising outcomes with good tolerability,^2)^ and a phase III trial demonstrated that SOX was as effective as cisplatin plus S-1 for advanced GC.^3)^ In addition, the ATTRACTION-4 study demonstrated that nivolumab combined with SOX or capecitabine plus oxaliplatin as first-line chemotherapy significantly improved progression-free survival.^4)^ Therefore, oral S-1-based chemotherapy is Japan’s standard and the most common first-line treatment for advanced GC.

However, patients with large Stage IV GCs invading the proximal stomach or the distal esophagus often presented with an inability to eat and drink. For those GC patients, the benefit of palliative gastrectomy (non-curative surgery) would be limited,^5)^ and bypass surgery such as gastro-jejunostomy is impossible to perform. In addition, GC patients find it difficult to receive oral S-1-based chemotherapy. A previous Japanese GC guideline suggested that evidence was lacking regarding chemotherapy for patients with no oral intake.^6)^ However, an elementary diet (ED) tube enables S-1-based chemotherapy as well as nutrition support for GC patients with an inability to eat and drink, but evidence of S-1-based chemotherapy using an ED tube for advanced GC patients with no oral intake is lacking. Therefore, we investigated the treatment results and the clinical benefit of S-1-based chemotherapy and nutrition support using an ED tube for patients with an inability to eat and drink due to a large Stage IV GC invading the proximal stomach in our institute.

## CASE PRESENTATION

### Patient population

This retrospective study was approved by the ethics committee at Chiba University Hospital (IRB number: HK202312-12). Written informed consent for the chemotherapy was obtained from all patients. However, due to its retrospective nature, written informed consent for participation in this study was substituted with a publicly posted disclosure document with an opt-out option. We retrospectively investigated the medical records of the patients with large Stage IV GCs invading the proximal stomach, leading to an inability to eat and drink, followed by S-1-based chemotherapy using an ED tube from June 2016 to March 2023. Patients with small bowel obstructions due to peritoneal metastasis were not included in this study.

### Placement of ED tube and enteral nutrition

**Fig. 1:**
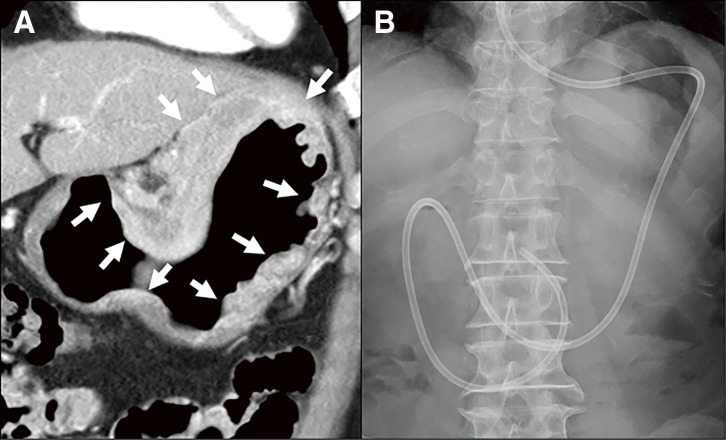
ED tube placement (**A**) Large type 4 gastric cancer invading not only the proximal stomach but the entire stomach. Arrows indicated tumor location. (**B**) For this patient, the ED tube was inserted via the nostril, and the leading edge of the tube was placed beyond the ligament of Treitz or at least the third portion of the duodenum. ED, elementary diet

Two types of ED tubes were used in this study. One was an 8 Fr or 10 Fr feeding tube (CORFLO feeding tube; NIPRO, Osaka, Japan), and another was a W-ED tube (Covidien, Tokyo, Japan) whose double-lumen structure allows for simultaneous enteral feeding and gastrointestinal tract decompression. ED tube was inserted via the nostril, and the leading edge of the tube was placed beyond the ligament of Treitz, or at least the third portion of the duodenum using a fluoroscopy or endoscopy (**Fig. 1**). This tube was used for not only S-1-based chemotherapy but also for enteral nutrition. Patients received 1400–1600 kcal of oligomeric formula (HINE E-GEL or Twinline-NF; Otsuka Pharmaceutical Factory, Inc., Tokyo, Japan) via ED tube.

### Treatment and follow-up

This study included three S-1-based chemotherapy regimens (SOX, SOX plus nivolumab, and SOX plus trastuzumab), and they have been detailed in previous studies.^2,4)^ Briefly, the SOX regimen was given every 3 weeks. S-1 (orally disintegrating tablet) 80 mg/m^2^ disintegrating with 20 ml plain hot water was provided via ED tube twice daily for 14 days, followed by a 1-week rest. On day 1, oxaliplatin 100 mg/m^2^ was intravenously administered. SOX plus nivolumab regimen was comprised of SOX regimen with intravenous administration of 360 mg nivolumab on day 1 of the cycle. Regarding the SOX plus trastuzumab regimen, in addition to the SOX regimen, trastuzumab was intravenously given at a dose of 8 mg/kg on day 1 of the first cycle, followed by 6 mg/kg every 3 weeks. These regimens were continued until disease progression, unacceptable toxicity, or patient refusal. Tumor responses were assessed by CT every 2–4 cycles of the chemotherapy. The tumor markers, including carcinoembryonic antigen and CA 19-9, were measured monthly. After these therapies, any additional treatment occurred at the discretion of the treating physician but basically followed Japanese gastric cancer treatment guidelines.^7)^ If a patient was considered a good responder to the chemotherapy based on the follow-up CT or clinical symptoms, we performed gastrography to check if the gastric stenosis was improved. If the stenosis was improved, the ED tube was removed, and the patient started oral food intake.

### Statistical analysis

Statistical analyses were carried out using the JMP Pro 16.0 (SAS Institute, Inc., Cary, NC, USA), and for all comparisons, *P* <0.05 was considered to indicate a statistically significant difference. Parameters of nutritional status at baseline and after the treatment were compared using the Wilcoxon signed-rank test. Kaplan–Meier analysis was performed for survival analysis, and the log-rank test was employed.

### Patient characteristics

A total of 20 patients (13 men and 7 women; median age 70 years) were eligible for this study. Seventeen patients were type 4, and 3 were type 3 GC, and the size of the main tumor was 13.15 ± 3.47 (8.7–21.9) cm, which was assessed by multiplanar reconstruction CT. These GCs were categorized as “large” GCs, according to a previous study.^8)^ Fifteen patients were treated with SOX, 4 were treated with SOX plus nivolumab, and one was treated with SOX plus trastuzumab because this patient had the HER2-positive GC. Two patients (10.0%) failed to complete even one cycle of the therapy due to severe nausea or diarrhea. The median follow-up time was 11.71 months. Patients’ characteristics are summarized in **Table 1**.

**Table 1:** Patient characteristics

Patient demographics	Variables	Value
Sex	Male/Female	13/7
Age	Median/range	70.0/47–84
Performance status	0/1/2	4/15/1
Histology	Diffuse/Intestinal	17/3
Tumor size (cm)	Mean ± SD (range)	13.15 ± 3.47 (8.7–21.9)
Tumor depth	cT4a/T4b	9/11
Lymph node metastasis	Positive/Negative	19/1
Distant metastasis (including peritoneal metastasis)	Positive/Negative	18/2
Peritoneal metastasis	Positive/Negative	15/5
The number of stage IV factors	Single/Multiple	11/9
Regimen	SOX/SOXN/SOXT	15/4/1

SOX, S-1 plus oxaliplatin; SOXN, SOX plus nivolumab; SOXT, SOX plus trastuzumab

### Treatment response, improvement of oral intake, and survival

**Fig. 2:**
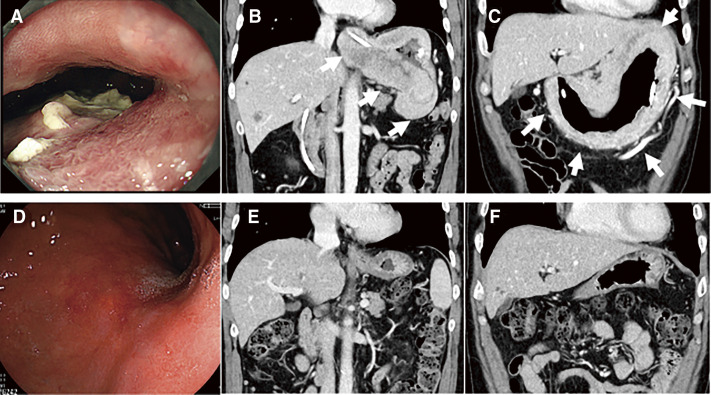
Case presentation. (**A**) Gastroscopy demonstrated large type 4 gastric cancer, and biopsy confirmed poorly differentiated adenocarcinoma. (**B**) and (**C**) were abdominal CT images (coronary view), and they showed that this huge tumor invaded not only the whole stomach but also the distal esophagus (arrows). This patient received S-1-based chemotherapy using an ED tube. The tumor shrank dramatically, and the ED tube was removed only after 2 weeks from the start of the treatment. After 1 year of the treatment, gastroscopy demonstrated the complete disappearance of the tumor (**D**), and no cancer cells were seen in the biopsy samples. Abdominal CT images after 1 year of the treatment also demonstrated the complete disappearance of the tumor (**E, F**). After 2 years of chemotherapy, this patient completed the chemotherapy and survived more than 80 months from the start of the chemotherapy without recurrence. ED, elementary diet

**Fig. 3:**
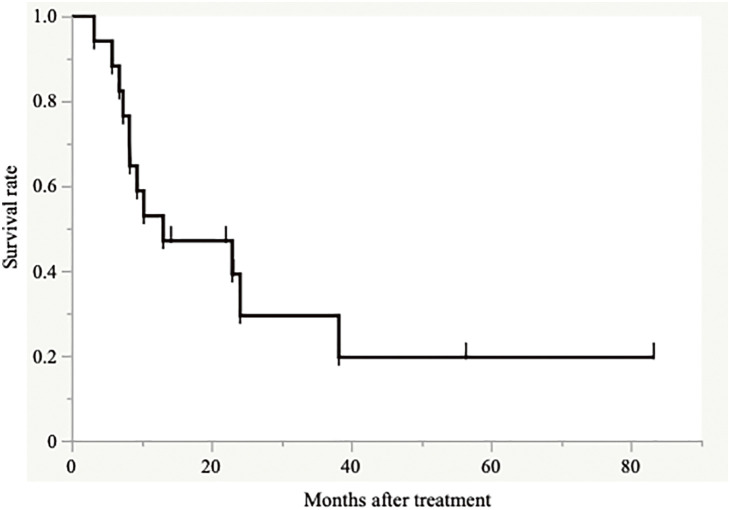
Disease-specific survival curve of 18 patients who could receive more than one cycle of S-1-based chemotherapy using Kaplan–Meier analysis.

Treatment response was evaluated in 18 patients who could receive more than one cycle of S-1-based chemotherapy, according to RECIST 1.1. One patient was diagnosed as complete response (CR), six were as partial response (PR), and five were as stable disease (SD) at their best responses, and the best response rate was 58.3%. A case showing an impressive response to the chemotherapy was demonstrated in **Fig. 2**. All of these 18 patients improved oral liquid intake after 47.5 ± 18.8 days (range: 14–79 days) from the start of the treatment, and 17 of those 18 patients improved oral intake of solid food after 54.5 ± 19.6 days (range: 15–84 days; 1–4 cycles of S-1-based chemotherapy), and ED tube was removed. One patient could not start oral intake of solid food because this patient died from a disease other than GC before starting oral solid food intake. All 18 patients were discharged from the hospital using enteral nutrition from the ED tube after 9 ± 18.5 days (range: 4–77 days) from the start of the chemotherapy. Three patients (16.7%) could receive conversion surgery after improvement of oral intake, and all cases received total gastrectomy (R0 resection, 2 cases; R1 resection, 1 case). Postoperative complication occurred in only one case (minor anastomotic bleeding), and postoperative hospital stay was 15–19 days. However, all patients who received conversion surgery died of cancer recurrence after 10.7–36.2 months of the surgery. The median disease-specific survival (DSS) of those 18 patients since the start of S-1-based chemotherapy was 13.1 months (**Fig. 3**).

### Changes in parameters of nutritional status and survival

Body weight, serum albumin level, and prognostic nutritional index (PNI) were compared between baseline and after about 1 month (1.129 ± 0.273, 0.7–1.433 months) of S-1-based chemotherapy and nutrition support using an ED tube (**Table 2**). During this period, body weight was significantly decreased (*P* = 0.0006), but serum albumin level and PNI were significantly improved (both *P* <0.0001). Univariate Cox regression analyses were performed to evaluate relationships between change rates of body weight, serum albumin level, and PNI during this period and DSS. Change rates of serum albumin level and PNI significantly correlated with DSS (*P* = 0.006, 0.01, respectively), whereas that of body weight did not (**Table 3**).

**Table 2:** Changes in parameters of nutritional status

	Baseline	After 1 month	*P*
Body weight	51.95 ± 9.23	47.7 ± 13.93	0.0006*
Serum albumin	2.98 ± 0.49	3.56 ± 0.31	<0.0001*
PNI	34.86 ± 5.34	41.75 ± 4.02	<0.0001*

*Significant difference at *P* <0.05. PNI, prognostic nutrition index

**Table 3:** Univariate Cox regression analysis of correlations of changes in nutrition markers and clinicopathological factors with disease-specific survival

Variables	HR	95% CI	*P*
Body weight change rate	0.975	0.950–1.006	0.06
Serum albumin change rate	1.069	1.018–1.128	0.006*
PNI change rate	1.062	1.009–1.130	0.01*
Age	1.032	0.951–1.120	0.4
Performance status (0 vs. 1–2)	1.188	0.314–4.484	0.7
Histology (Diffuse vs. Intestinal)	0.577	0.072–4.578	0.5
Peritoneal metastasis (Positive vs. Negative)	0.48	0.140–1.674	0.27
The number of stage IV factors (Single vs. Multiple)	0.78	0.234–2.624	0.6
Conversion surgery (Positive vs. Negative)	0.80	0.209–3.061	0.7

*Significant difference at *P* <0.05. PNI, prognostic nutrition index

## DISCUSSION

The population of patients with large Stage IV GCs invading the proximal stomach, leading to no oral intake, might be limited. However, in clinical practice, we are sometimes asked to treat such patients. Treatment strategy for those GC patients has not been standardized yet due to a lack of evidence.^6)^ A systematic review suggested that the benefit of palliative gastrectomy would be limited because of no significant effect on long-term survival and significantly increased overall surgical complications.^5)^ Regarding systemic chemotherapy for Stage IV GC, oral S-1-based chemotherapy is the standard and the most effective first-line treatment for advanced GC in Japan. However, oral chemotherapy is impossible for patients who have GCs invading the upper stomach or lower esophagus, leading to no oral intake. In this context, a few previous case reports demonstrated the feasibility of S-1-based chemotherapy using the nasojejunal tube for advanced GC.^9–11)^ Therefore, we started S-1-based chemotherapy using an ED tube for such GC patients who were unable to eat and drink orally. However, the treatment outcome of S-1-based chemotherapy using an ED tube for large Stage IV GC patients with an inability to eat and drink has not been reported in a cohort study with a certain number of patients.

In this study, we investigated the clinical outcome of large Stage IV GC patients with oral intake inability who received S-1-based chemotherapy using an ED tube. Interestingly, our study demonstrated that 94% of patients who could receive more than one cycle of S-1-based chemotherapy improved their oral intake of solid food within 3 months. If we excluded one patient who died from a disease other than GC before starting oral solid food intake, 100% of patients could improve oral intake, which was really impressive data. Considering that this study cohort included 85% of type 4 Stage IV GC, the median DSS of 13.1 months might be favorable. This DSS might be comparable to the survival data of GC patients with gastric outlet obstruction treated with palliative gastrectomy or gastrojejunostomy, which was reported by Terashima et al.^12)^ Given such favorable outcomes of S-1-based chemotherapy and nutrition support using an ED tube, it might be better not to choose palliative total gastrectomy with a risk of non-curative resection and surgical complications. Regarding intravenous chemotherapy for GC patients with an inability to eat, Shitara et al.^13)^ reported that 40% of patients achieved improvement in oral intake with a median duration of nutritional-support-free time of 3.1 months in 2010. Currently, mFOLFOX6 might be the strongest intravenous chemotherapy for GC with a median overall survival of 11.3 months,^14)^ but this median survival was worse than the SOX regimen.^3)^ The data on the pharmacokinetics of S-1 administration via ED tube were limited, but a few papers suggested that plasma concentration levels of S-1 were equivalent to oral intake, and no severe adverse effects, particularly on the digestive systems, were observed.^15,16)^ In addition, our study demonstrated that improvement in nutrition status, such as serum albumin level and PNI, was significantly associated with better survival. It suggested the prognostic importance of nutrition in the treatment of advanced GC. In fact, Karabulut et al.^17)^ reported that malnutrition was obviously associated with poor survival in chemotherapy of metastatic GC patients. However, body weight was significantly decreased in this cohort during the first 1 month from the start of S-1-based chemotherapy, though significant improvement of serum albumin level and PNI was observed. Possible reasons for this discrepancy might be as follows: (1) Patients finally received 1400–1600 kcal of oligomeric formula via ED tube, but they started their nutrition support via ED tube in a gradual manner; therefore, they could not get enough nutrition for first 1–2 weeks in this period, leading to body weight loss. (2) Poor nutritional status due to poor oral intake before nutrition support via ED tube might have an effect on following body weight loss. In terms of nutrition support, it was reported that enteral nutrition was superior to parenteral nutrition in the treatment of advanced cancer.^18)^ Therefore, S-1-based chemotherapy plus enteral nutrition using an ED tube might be the best treatment strategy for GC patients with an inability to eat, rather than palliative surgery and intravenous chemotherapy.

Our study has limitations as follows. First, this study is based on single-center data, and the sample size is small. Second, the retrospective nature of this study is also a limitation of this study. A prospective multicenter study with a larger patient population is desirable, but the population of patients with large Stage IV GCs invading the proximal stomach leading to no oral intake would be quite limited, which might be an obstacle to conducting such a large prospective study.

## CONCLUSION

Given the high success rate of improvement in oral intake, S-1-based chemotherapy using an ED tube can be a promising treatment option for large Stage IV GC with an inability to eat and drink, even though this is a retrospective study with a small sample size.

## ACKNOWLEDGMENTS

The authors express gratitude to all the staff involved in the cases of this study.

## DECLARATIONS

### Funding

The authors received no funding support for this research.

### Authors’ contributions

KH and YK conceived and designed this study.

KH wrote the main manuscript text and prepared all tables and figures.

KH, YK, and GO worked for ED tube insertion via the nose.

KH, YK, YM, RO, NS, TT, AN, TS, MU, GO, and HM treated patients and analyzed the patient data.

All authors read and approved the manuscript.

### Availability of data and materials

The data that support the findings of this study are available from the corresponding author upon reasonable request.

### Ethical approval and consent to participate

This retrospective study was approved by the ethics committee at Chiba University Hospital (IRB number: HK202312-12). Written informed consent for participation in this study was substituted with a publicly posted disclosure document with an opt-out option due to the retrospective nature of this study. All procedures performed in this study were in accordance with the Helsinki Declaration.

### Consent for publication

Informed consent for publication was obtained from the patients via the opt-out method, owing to the retrospective nature of this study, with no risk to the participants. However, written informed consent was obtained from the patient regarding the usage of medical imaging for this publication.

### Competing interests

The authors declare that they have no competing interests.
